# SARS-CoV-2 incidence among teaching staff in primary and secondary schools—Wales, 2020–2021

**DOI:** 10.1186/s12889-023-15680-1

**Published:** 2023-05-19

**Authors:** Ffion Thomas, André Fedeli, Emily Steggall, Jose Maria Gonzalez Gonzalez, Jane Salmon, Christopher Williams, Noel Craine

**Affiliations:** 1 Observatory and Cancer Analysis Team, Public Health Wales, 2 Capital Quarter, Tyndall St, CF10 4BQ Cardiff, UK; 2 School of Medicine, Cardiff University, CF14 4XN Cardiff, UK; 3 Communicable Disease Surveillance Centre, Public Health Wales, Capital Quarter, Tyndall St, CF10 4BQ Cardiff, UK

**Keywords:** COVID-19, Communicable disease, Education, Control, Public Health

## Abstract

**Background:**

During the COVID-19 pandemic, face-to-face delivery of education in schools across Wales was disrupted with repeated school closures to limit risk of infection. Evidence describing the incidence of infection amongst school staff during times when schools were open is limited. A previous research study found infection rates were higher in English primary school settings when compared with secondary. An Italian study suggested teachers weren’t at greater risk of infection in comparison to the general population. The aim of this study was to identify whether educational staff had higher incidence rates than their counterparts in the general population in Wales, and secondly whether incidence rates amongst staff differed between primary and secondary school settings and by teacher age.

**Methods:**

We performed a retrospective observational cohort study using the national case detection and contact tracing system implemented during the COVID pandemic. Age stratified person-day COVID-19 incidence rates amongst teaching staff linked to primary or secondary schools in Wales were calculated for the autumn and summer terms during 2020–2021.

**Results:**

The observed pooled COVID-19 incidence rates for staff across both terms was 23.30 per 100,000 person days (95% CI: 22.31–24.33). By comparison, the rate in the general population aged 19–65, was 21.68 per 100,000 person days (95%: CI 21.53–21.84). Incidence among teaching staff was highest in the two youngest age groups (< 25 years and 25–29 years). When compared to the age matched general population, incidence was higher in the autumn term amongst primary school teachers aged ≤ 39 years, and in the summer term higher only in the primary school teachers aged < 25 years.

**Conclusion:**

The data were consistent with an elevated risk of COVID-19 amongst younger teaching staff in primary schools when compared to the general population, however differences in case ascertainment couldn’t be excluded as a possible reason for this. Rate differences by age group in teaching staff mirrored those in the general population. The risk in older teachers (≥ 50 years) in both settings was the same or lower than in the general population. Amongst all age groups of teachers maintaining the key risk mitigations within periods of COVID transmission remain important.

**Supplementary Information:**

The online version contains supplementary material available at 10.1186/s12889-023-15680-1.

## Background

The World Health Organization declared COVID-19 a pandemic in March 2020, [2]. At this time, and until September 2021, face-to-face delivery of education in schools across Wales was disrupted with repeated Welsh Government mandated partial and full school closures. Additionally, positive cases and contacts of cases were excluded from school [3].

Globally, COVID-19 outbreaks have been documented within school settings, but rates of infections appear to be low when there are appropriate mitigation measures in place, [4]. In Wales, many community level mitigation measures were advised including physical distancing, self-isolation and infection control measures, and schools received guidance on how to minimise COVID-19 risk. Little is known about the effectiveness of these measures within the school setting and risk to school staff in Wales. However, an English study found higher infection rates in primary school staff compared to secondary school staff. Within primary school settings the wearing of face masks was discretionary for staff, whereas staff and pupils in secondary schools were advised to wear face coverings in communal areas where physical distancing was difficult to maintain [5].

Two key public health questions with respect to the transmission of COVID-19 in schools are firstly, whether educational staff were had higher rates of incidence than their counterparts in the general population in Wales, and secondly whether incidence rates amongst staff differed between primary and secondary school settings. These questions are assessed in this paper by comparing incidence rates within school teaching staff across school settings in Wales during two periods when schools were open to pupils. We compared these rates to those observed in the general population. We addressed this question using data collected as part of the national case and contact tracing system that allowed linking of COVID-19 cases to place of employment.

## Methods

### Study design and setting

The study was a retrospective cohort study with locations included in surveillance being maintained primary and secondary schools in Wales. Primary schools include pupils aged 3–11 and secondary schools include pupils aged 11–19.

**Fig. 1:**
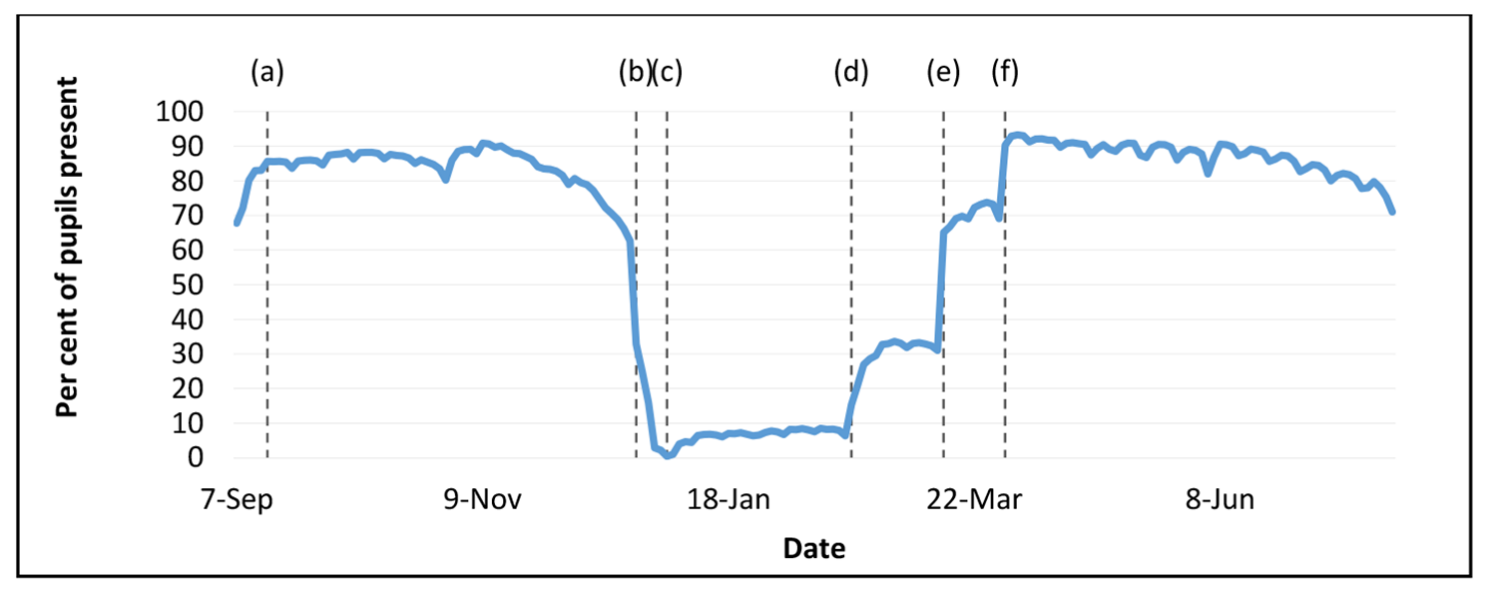
**(a)** all schools re-opened, **(b)** all secondary schools move to remote learning and most primary schools close for Christmas, **(c)** schools in Wales were open only to vulnerable pupils or pupils of key workers, **(d)** phased return of foundation phase, **(e)** all remaining primary aged pupils and those in qualification years return to learning on site, **(f)** all remaining pupils return to learning on site. Source: Welsh Government Attendance Data

The number of pupils attending schools varied over the course of the pandemic, we thus looked at two surveillance periods during which school attendance was broadly stable and above 75% in both sectors (Fig. 1). We assumed during these periods schools would be seeking to staff at full capacity whilst recognising normal and COVID-19 related staff absences. The first period covered the autumn school term (14th September 2020 to 11th December 2020), and the second period covered the summer term (12th April 2021 to 16th July 2021).

### Participants and data sources

The participants included Welsh residents classified as primary or secondary school teaching staff. Cases were identified using the Test, Trace and Protect (TTP) database. This database was created through the contact tracing process of COVID-19 cases in Wales, in which the TTP service would collate data on PCR positive cases via phone calls. Data collected included personal information, workplace address and exposure history such as contacts and places visited. Denominator data on the number of school staff was acquired from the School Workforce Annual Census data 2020/21 (SWAC).

To allow for comparison to the denominator data, we only included cases where the job role recorded in the TTP dataset matched the SWAC roles (supplementary material A). Further inclusion criteria included being recorded between ages 19–65, and where either the employment location was listed as a maintained primary or secondary school in Wales or attending the setting during the appropriate school term along with any of the following: listed as a teacher, relation to the school was work, classified as a key worker in Education and Childcare.

Cases had to be successfully interviewed for the TTP database for us to identify them as being linked to the school via their workplace address or exposure history. Therefore, all cases within the study were interviewed. It is likely that we missed some cases amongst both teachers and the general population due to failure to test, false negative tests, or failure to contact TTP. We assume that this lack of ascertainment was not biased to any particular age or employment group. If such a bias did exist and for example cases were more likely to be detected amongst teaching staff than the general population then this would introduce error into the estimates we made.

An age-matched general population group was used to compare rates in teaching staff to the rates in the general population in Wales. The Office of National Statistics population estimates were used to obtain population denominator data. The Welsh Laboratory Information Management System was used to determine case numbers within the general population of Wales.

### Data quality

The TTP database was developed and introduced as part of the national COVID-19 response. Over the course of the study period additional information was continually collected and TTP data collection guidance changed accordingly.

### Validation of cases reported in teaching staff

To validate the consistency across Wales of reporting of staff cases in the TTP database we compared, within each local authority, the rate observed in the community to that reported in teaching staff. We assumed that cases amongst staff would broadly reflect cases within the local community, as staff both form part of the local community and are exposed to a similar level of background risk. We recognise however that true outbreaks may occur within teaching staff. We then generated scatter plots for each local authority of staff cases vs. community cases, with each point representing the case rates for a fortnightly period and applied a line of best fit to estimate reporting consistency across local authorities.

### Data analysis

Incidence rates were used because they allowed for comparison of different settings and time periods. This enabled us to compare primary settings to secondary settings, and autumn to summer term. We used two measures of incidence: person-days incidence and age standardised incidence.

Person-days incidence rates were used to allow for the comparison of rates at time periods of different lengths and different settings, and 95% confidence intervals (CIs) were calculated.

The person-day rates ($$ r)$$ were calculated by

1 $$ r=\frac{O}{n}\times \text{100,000}$$

where:

*O* = number of confirmed COVID-19 cases in each group.

*n* = denominator population-days at risk.

The denominator was calculated by

2 $$ n=\frac{number at risk}{days in period}$$

Where the number at risk was the number of full-time teaching staff registered at each school (irrespective whether staff were onsite on any one day) divided by the number of days of exposure. 95% CIs were calculated using Byar’s method, [6].

Age standardised incidence rates were estimated using direct age standardisation. This was done to compare overall rates for both time periods in staff to those in the age-matched population, and to compare rates in primary school staff to those in secondary school staff. The Wales population was used as the reference population and to generate age-specific rates (Table 1). Direct age standardisation used incidence proportion, rather than incidence rate, and is therefore reported per 10,000 population (rather than 100,000 person days). The same method was used to calculate a crude incidence (per 10,000 population) for the age-matched Wales population.

## Results

Case numbers and incidence varied by setting and age (Table 1). Overall person-days incidence was lower in staff and in the general population in the summer term than in the autumn term. In the autumn term 1,735 cases were observed in teaching staff, in comparison to 337 cases in the summer term. The observed pooled incidence rates for staff across both time periods was 23.30 per 100,000 person days (95% CI; 22.31–24.33). By comparison, the rate in the general population (aged 19–65) for the same time periods was 21.68 per 100,000 person days (95% CI; 21.53–21.84).

Age standardised incidence for staff across both periods was 224.70 per 10,000 population (95% CI; 213.65-236.11). By comparison, the crude rate in the general population (aged 19–65) for the same time periods was 198.41 per 10,000 population (95% CI; 196.98-199.84). Age standardised incidence for primary school staff across both periods was 245.46 per 10,000 population (95% CI; 231.08-260.42) and for secondary school staff 188.70 per 10,000 population (95% CI; 171.93-206.49) for the same time periods.

**Fig. 2:**
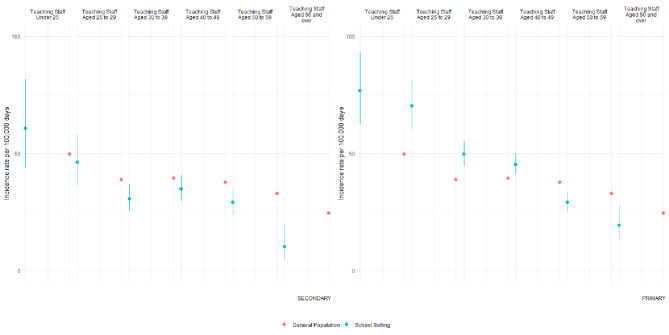
Autumn Term Incidence rate with 95% CIs in teaching school staff by school sector and age strata, and the incidence rate in the Welsh population equivalent age strata. Rates calculated for Autumn Term; 14th September 2020–11th December 2020

**Fig. 3:**
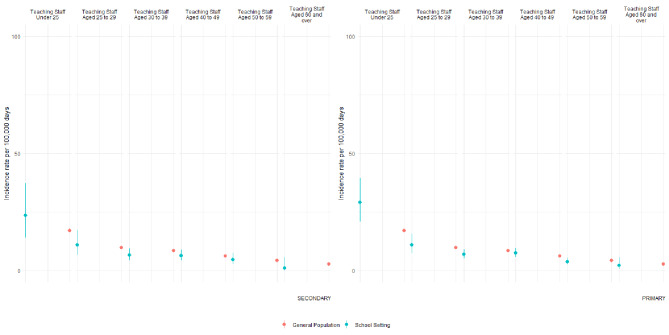
Summer Term Incidence rate with 95% CIs in teaching school staff by school sector and age strata, and the incidence rate in the Welsh population equivalent age strata. Rates calculated for Summer Term; 12th April 2021–16th July 2021

Throughout both terms the data suggested a general downward trend in incidence per 100,000 person days with increasing age in both teaching staff and the general population (Figs. 2 and 3). In the autumn term, a period of high background COVID-19 incidence in Wales, incidence amongst staff in primary schools in younger age groups (25–29, 30–39 and 40–49 years) was higher than that reported amongst their age-matched counterparts in the general population. In secondary schools, we observed a lower incidence amongst staff in age groups 30–39 and 60 + than their counterparts in the general population. When comparing across school sectors the data from the autumn term, the period of higher community transmission, suggested that amongst younger teachers risk may have been higher within primary schools than secondary schools.

During the summer term, when background COVID-19 incidence was lower than in the autumn term, again the observed incidence rates followed a similar trend to the rates observed in the age-matched general population. When comparing staff to their counterparts in the general population an elevated incidence was seen only amongst primary staff aged < 25 years.

**Table 1:** Denominator and case data with estimated incidence by school sector, age group and term

Term	Population	Setting	Age Group	Denominator	Cases	Incidence (per 100,000 person days)	95% UCI	95% LCI
Autumn	Teaching Staff	Primary School	19 to 24	1480	100	76.78	93.39	62.47
			25 to 29	2875	178	70.36	81.49	60.40
			30 to 39	7745	338	49.59	55.17	44.45
			40 to 49	9460	377	45.29	50.10	40.83
			50 to 59	7540	193	29.09	33.49	25.13
			60 to 65	1930	33	19.43	27.29	13.37
		Secondary School	19 to 24	805	43	60.70	81.77	43.92
			25 to 29	1820	74	46.20	58.01	36.28
			30 to 39	4555	123	30.69	36.61	25.50
			40 to 49	5345	164	34.87	40.63	29.73
			50 to 59	4035	103	29.01	35.18	23.68
			60 to 65	1005	9	10.18	19.32	4.64
	General Population	Community	19 to 24	240,130	10,481	49.60	50.56	48.65
			25 to 29	208,260	7106	38.77	39.69	37.88
			30 to 39	377,874	13,065	39.29	39.97	38.62
			40 to 49	368,628	12,217	37.66	38.34	37.00
			50 to 59	437,960	12,677	32.89	33.47	32.32
			60 to 65	230,003	4944	24.43	25.12	23.75
Summer	Teaching Staff	Primary School	19 to 24	1480	41	29.16	39.56	20.92
			25 to 29	2875	30	10.98	15.68	7.41
			30 to 39	7745	51	6.93	9.11	5.16
			40 to 49	9460	68	7.57	9.59	5.88
			50 to 59	7540	27	3.77	5.48	2.48
			60 to 65	1930	4	2.18	5.59	0.59
		Secondary School	19 to 24	805	18	23.54	37.20	13.94
			25 to 29	1820	19	10.99	17.16	6.61
			30 to 39	4555	28	6.47	9.35	4.30
			40 to 49	5345	32	6.30	8.90	4.31
			50 to 59	4035	18	4.70	7.42	2.78
			60 to 65	1005	1	1.05	5.83	0.01
	General Population	Community	19 to 24	240,130	3908	17.13	17.68	16.60
			25 to 29	208,260	1945	9.83	10.28	9.40
			30 to 39	377,874	3013	8.39	8.70	8.10
			40 to 49	368,628	2182	6.23	6.50	5.97
			50 to 59	437,960	1767	4.25	4.45	4.05
			60 to 65	230,003	615	2.81	3.05	2.60

### Validation

Educational staff cases broadly followed community rates in 19/22 local authorities for secondary schools, and 22/22 local authorities in primary schools. Whilst there may be some variation in the quality of data reported in the TTP database over time and geography these data suggest reasonably consistent reporting across Welsh local authorities. This provides confidence in the validity of the educational staff data.

## Discussion

The analysis of cases amongst school staff in Wales, for two time periods when schools were open to pupils suggests that, overall, the incidence rate of COVID-19 in school educational staff was slightly higher than in the age matched general community. Results of age-standardisation revealed that this observed difference could not be explained by any age differences in the two populations. However, comparing rates in age strata separately for both time periods and different settings (primary schools, secondary schools and the general population), indicated that the higher overall incidence was explained by higher rates in younger staff in the primary school setting. As increasing age is a risk factor for poorer COVID-19 outcomes, [1], an important finding was that older staff (age 50+) didn’t have a higher risk than the age-matched population. This suggests that non-pharmaceutical measures in older teachers in these settings were effective. A similar pattern across age strata was observed amongst staff and the wider community indicating a decreasing incidence with increasing age. An Italian study comparing infection rates in teachers to their counterparts in the general population found that teachers weren’t at increased risk of infection, [7]. A Norwegian study found that teachers weren’t at increased risk in the first wave but had a moderate increased risk during the second wave, [8]. It’s important to recognize that ascertainment of infection may have differed between teaching staff and the general population, and potentially between different age groups and between school settings.

Detected case rates will have been influenced by policy on testing for COVID-19. In February 2021 all teaching staff and school pupils in years 10–13 (aged approximately 15–19) were encouraged to self-test twice a week using lateral flow testing kits (LFTs), this was extended on 19th April 2021 to all school pupils in years 7–9 (aged approximately 11–14), [3]. Following a positive LFT, individuals were then prompted to have a confirmatory PCR. Similar regular asymptomatic screening wasn’t recommended for the general population and was generally restricted to specific occupational settings. Ascertainment of asymptomatic infection initially by LFT and confirmed by PCR was thus likely to have been higher amongst teaching staff than the general population, this is likely to have been higher for the summer term following the policy change.

The methods used in our study don’t allow us to draw firm conclusions around differential risk between school sectors. However, after direct age standardisation, rates in primary school staff were found to be higher than in secondary school staff. The different rates observed in primary and secondary school staff supports findings from a study in England, [5]. However it’s plausible that exposure to COVID-19 varied by school setting with the data suggesting higher exposure in primary school settings amongst younger staff than the same age groups in secondary schools during the autumn term (a period of higher overall community transmission).

Younger children with COVID-19 are more likely to be asymptomatic, so can only be identified via testing, [9]. However, during the time period examined in this study there was no encouragement of LFT testing for primary school children, perhaps leading to less case ascertainment and lower subsequent isolation of positive cases and thus higher exposure to staff. Fewer mitigation measures were in place within primary schools in comparison to secondary schools. Although all schools were encouraged to maintain social distancing (2 m) where possible to reduce the risk of viral transmission, this wasn’t always possible when working with younger pupils, [3]. Throughout the summer term, face coverings became mandatory in all secondary school areas outside of the classroom for staff and pupils to lower the risk of transmission, but only for staff in primary schools, [3]. Inconsistent mask wearing could contribute to school-based outbreaks among staff, [10, 11]; we did not however access any data on mask wearing by staff in Welsh schools for the periods under consideration.

The established increase in the risk of severe illness from COVID-19 with age, [12] may have impacted social mixing patterns within and outside of the school setting. We don’t know the extent to which age may have impacted on attitudes to risk amongst teaching staff in Wales. We didn’t examine data on other risk factors that may have varied with teacher age outside of the work setting (for example use of public transport, house sharing, and extent of social mixing). In secondary school settings, staff-to-staff transmission is more common than transmission from students to staff, staff to students, or student to student, [13, 11]. Little research has been done to explore this relationship in primary schools. However, our observations suggest teaching staff aged < 25 were more at risk than the age-matched general population, suggesting transmission within the school setting is plausible in this age group.

Vaccination uptake amongst school aged children may have influenced the risk of exposure to teaching staff in the summer term. Vaccination began in Wales on 8th December 2020, by 5th April those in higher priority groups had received their first vaccine which included anyone 50+, 16–64 year-olds with underlying health conditions, and frontline health and social care workers, [3]. Those aged 40–49 began to receive their vaccine from 20th April, 30–39 year-olds from the 25th May and 18–29 year olds from 8th June. The protective effects of the vaccine may be evident by the lower positivity rates in the summer term when compared with the autumn, in school settings and the general population.

### Limitations

The use of LFTs twice weekly in secondary schools may have improved ascertainment of asymptomatic cases in comparison to other sectors. As the testing policy changes took place between the two periods examined in this study, they may have resulted in more asymptomatic cases linked to educational settings in the later period. Furthermore, there may have been differences in application of TTP protocols across time and by geography within Wales influencing data quality.

Our estimates don’t control for ascertainment bias that may vary by setting and age. Furthermore, comparing teaching staff to the general population may not be valid, even when looking at specific age strata. Transmission in the school setting is difficult to assess; identifying a case as attending a school setting does not necessarily mean that the transmission occurred within the school.

We did not seek to determine formal statistical difference between the populations but rather showed estimates with 95%CIs. This allowed an assessment of the precision around the estimates without invoking an assumption of statistical validity that’s not warranted by this study design.

Changes in TTP data guidance may have impacted data completeness. The TTP data collection relies on cases providing accurate information about their exposure history including whether they have been exposed to the school setting and is therefore subject to self-reporting bias.

There may be occasions where teaching cases were identified via their workplace address, but they may not have entered the school during their infectious period. In addition, confirmed cases resident in England or with an unknown/missing/invalid post code were not included in this report. Such individuals don’t appear in the TTP database yet may be linked to a school in Wales.

The TTP database developed to manage a COVID-19 contact tracing system can provide insight into disease incidence, however, possible ascertainment bias and variation in the reporting coverage over time and potentially by geography should be considered when interpreting findings.

## Conclusion

Incidence of COVID-19 observed amongst teaching staff was slightly higher than observed in the general population. Cases detected amongst school staff followed similar trends by age and time to community rates. A higher incidence of COVID-19 in staff was observed in all educational settings in the autumn term than in the summer term, which reflected levels of transmission in the community during those periods. Amongst all age groups of teachers, maintaining the key risk mitigations within periods of COVID transmission remain important.

The data suggested a higher incidence rates amongst younger staff in primary schools, apparent in those aged < = 39 in the autumn term and < 25 in the summer term, when compared to the aged matched general population. Rates amongst older staff (50+) were generally equal to or less than the aged match population. However, we don’t know whether this reflects a true increase in risk or different case ascertainment in staff as compared to the general population. When comparing primary school staff to secondary school staff for both periods combined, a higher age-adjusted rate of infection was observed in primary school staff overall than in secondary school staff. Age specific rates by setting varied in the Autumn term, where higher rates were observed in primary teaching staff aged 25 to 49 than in the same secondary teaching staff age groups.

## Electronic supplementary material

Below is the link to the electronic supplementary material.

Supplementary Material 1

## Data Availability

The Welsh Laboratory Information Management System and the Test, Trace and Protect (TTP) database were used to determine relevant case numbers. These data management systems not in the public domain but were accessed as part of the statutory functions of Public Health Wales relating to surveillance of infectious disease. Denominator data on the number of school staff was acquired from the School Workforce Annual Census data 2020/21 (SWAC). The Office of National Statistics population estimates were used to obtain population denominator data. The datasets generated and analysed during the current study are not publicly available due to confidentiality but are available from the corresponding author on reasonable request.
