# Supplementary material for: SARS-CoV-2 incidence among teaching staff in primary and secondary schools—Wales, 2020–2021
Source: BMC Public Health. 2023 May 19;23:922. doi: 10.1186/s12889-023-15680-1 (PMC10197026; doi:10.1186/s12889-023-15680-1)
Supplement: Supplementary file 1 — Supplementary Material 1 [file 12889_2023_15680_MOESM1_ESM.docx]

**Supplementary material A**

**Staff job roles listed in the TTP database that were included under the ‘Teaching Staff’ classification.**
